# Artificial Intelligence-Assisted ACR TI-RADS Assessment of Thyroid Nodules: Potential for Reducing Unnecessary Biopsies in a Prospective Single-Center Study

**DOI:** 10.3390/tomography12100144

**Published:** 2026-10-01

**Authors:** Rojda Kotan, Bahri Evren

**Affiliations:** Department of Endocrinology and Metabolism, Faculty of Medicine, Inonu University, 44280 Malatya, Turkey; bahri.evren@inonu.edu.tr

**Keywords:** artificial intelligence, thyroid nodules, TI-RADS, fine-needle aspiration, unnecessary biopsy, thyroid cancer

## Abstract

Background: Artificial intelligence (AI)-based ultrasound risk stratification has emerged as a promising tool for improving the evaluation of thyroid nodules. However, prospective evidence regarding its performance in routine clinical practice and its potential impact on biopsy recommendations remains limited. Methods: In this prospective single-center observational study, 1009 thyroid nodules undergoing ultrasound-guided fine-needle aspiration between May 2025 and May 2026 were independently evaluated using ChatGPT Plus as an investigational AI-assisted decision-support tool. Representative ultrasound images, including dimensional and Doppler images, were submitted to the AI without disclosure of the radiologist-assigned TI-RADS category or subsequent cytological or histopathological findings. AI-assisted and radiologist-assigned TI-RADS classifications were compared with Bethesda cytology, while postoperative histopathology served as the reference standard in the surgically treated subgroup. Diagnostic performance and discordance in risk classification between AI and routine radiologist assessment were evaluated. Results: The study included 1009 thyroid nodules from patients with a mean age of 51.5 ± 13.4 years, of whom 80.7% were female. Bethesda II was the most frequent cytological category (93.6%). AI classified 227 nodules (22.5%) as high risk (TR4–TR5), compared with 761 nodules (75.5%) by routine radiologist assessment (*p* < 0.001). Among Bethesda II nodules, AI assigned 172 (18.2%) to TR4–TR5 compared with 701 (74.2%) by radiologists. When ACR TI-RADS category-specific size thresholds were applied, FNA would have been recommended for 469 nodules (46.5%) according to AI-assisted classification and 993 nodules (98.4%) according to radiologist-assigned classification (*p* < 0.001), corresponding to 524 fewer modeled FNA recommendations with AI-assisted assessment. Histopathological evaluation was available for 73 surgically treated nodules, including 48 malignant and 25 benign lesions. Within this surgically verified subgroup, both AI-assisted and radiologist-assigned TI-RADS achieved a sensitivity of 100.0%, while specificity was 72.0% for AI-assisted assessment and 60.0% for radiologist assessment. Because histopathological verification was available only for surgically treated nodules, these diagnostic performance estimates apply specifically to the surgically verified subgroup and cannot exclude false-negative disease among nonoperated nodules. Conclusions: AI-assisted ACR TI-RADS assessment produced substantially fewer high-risk assignments than routine radiologist assessment, with the greatest divergence observed among cytologically benign nodules. These findings suggest a potential role for AI as a second-reader decision-support tool for refining thyroid nodule risk stratification and biopsy selection. However, because all nodules had already undergone FNA and histopathological verification was limited to surgically treated cases, the present study cannot establish the safety of AI-guided biopsy de-escalation or exclude false-negative disease among nonoperated nodules. Prospective management studies with longitudinal outcome verification are required to determine whether AI-assisted assessment can safely reduce unnecessary FNA.

## Simple Summary

Artificial intelligence may help improve the consistency of ultrasound-based risk assessment of thyroid nodules and support decisions about which nodules require biopsy. In this prospective single-center study, we compared artificial intelligence-assisted American College of Radiology Thyroid Imaging Reporting and Data System (ACR TI-RADS) assessment with routine radiologist assessment in 1009 thyroid nodules that underwent fine-needle aspiration. The artificial intelligence-assisted approach classified substantially fewer nodules as high risk, with the greatest difference observed among nodules with benign cytology. In the subgroup of surgically treated nodules, all malignant lesions were identified as high risk by the artificial intelligence-assisted assessment. However, because all nodules in this study had already undergone biopsy and only a subset had surgical confirmation, our findings do not demonstrate that biopsies can safely be avoided in clinical practice. Rather, they suggest that artificial intelligence may have a role as a second-reader decision-support tool to refine thyroid nodule risk assessment and biopsy selection. Further prospective studies with broader populations and long-term follow-up are needed before this approach can be incorporated into routine clinical practice.

## 1. Introduction

Thyroid nodules are among the most common endocrine findings encountered in clinical practice, and their detection has increased substantially with the widespread use of high-resolution ultrasonography and cross-sectional imaging. The central clinical challenge is not simply to identify nodules but to distinguish the minority that require invasive investigation or treatment from the much larger group that can be managed conservatively. Because most thyroid nodules are benign, a strategy that maximizes cancer detection at the expense of very low specificity may lead to repeated imaging, fine-needle aspiration (FNA), indeterminate cytology, and potentially avoidable surgery. Contemporary international guidelines therefore emphasize risk-adapted management in which sonographic appearance, nodule size, clinical context, and selective FNA are integrated rather than applying biopsy uniformly to all detected nodules [1,2,3,4].

Ultrasonography is the primary imaging modality for thyroid nodule evaluation because it is widely available, non-ionizing, and capable of characterizing features associated with malignancy. Several standardized ultrasound risk-stratification systems, including ACR TI-RADS, EU-TIRADS, and K-TIRADS, have been developed to translate combinations of sonographic findings into clinically meaningful risk categories and biopsy thresholds [5,6,7,8,9]. These systems have improved reporting consistency and provide a common language for radiologists, endocrinologists, surgeons, and cytopathologists. Nevertheless, the classification process still depends on human recognition and interpretation of features such as composition, echogenicity, margins, shape, and echogenic foci. Differences in operator experience and in the weighting of borderline features can therefore shift the same nodule between risk categories and alter whether FNA is recommended [10,11,12,13].

The consequences of this variability are particularly relevant in a high-volume thyroid clinic. A false-positive high-risk ultrasound classification may not directly cause harm, but it can initiate a cascade of additional procedures. FNA is generally safe, yet it is invasive, consumes clinical and cytopathology resources, may cause discomfort or anxiety, and can produce nondiagnostic or indeterminate results that require repeat biopsy, molecular testing, surveillance, or diagnostic surgery. The 2023 Bethesda System has improved standardization of cytological reporting and malignancy-risk estimates, but cytology cannot eliminate the upstream problem of selecting which nodules should undergo biopsy in the first place [3,14,15,16,17,18]. Consequently, improving specificity at the ultrasound triage stage, while preserving very high sensitivity for clinically relevant malignancy, represents an important opportunity to reduce over-investigation.

Artificial intelligence (AI) is increasingly being investigated as a means of addressing this problem. Computer-aided and deep learning systems can evaluate sonographic patterns in a reproducible manner, provide malignancy estimates, and support assignment of TI-RADS categories. Published studies have reported diagnostic performance comparable with experienced readers in selected settings, and AI-assisted approaches may be especially useful for reducing variability between operators and standardizing interpretation across institutions [6,11,19,20,21,22,23,24,25,26]. However, diagnostic accuracy alone is not sufficient to establish clinical utility. A model with a favorable area under the receiver operating characteristic curve may still have limited practical value if it does not change management, if its performance depends heavily on image acquisition, or if improved specificity is achieved at the cost of missed cancers.

Accordingly, a clinically useful thyroid ultrasound AI system should be evaluated not only against a reference diagnosis but also in relation to the decisions that matter in routine care. Recent work has increasingly focused on whether AI can refine biopsy selection and reduce unnecessary FNA. Decision-support systems have shown the potential to decrease biopsy recommendations and improve efficiency, while systematic reviews emphasize the need for prospective and real-world validation before broad implementation [7,8,9,23,24,25]. This distinction is important because many published datasets are retrospective, enriched for surgically confirmed malignancy, or assembled under experimental conditions that may not reflect the distribution of nodules seen in everyday practice.

Taken together, the available evidence suggests that AI may function most appropriately as a clinical decision-support tool rather than as an autonomous replacement for radiological or endocrinological judgment. Its potential advantages include reproducible feature assessment, consistent risk categorization, and a reduction in unnecessary invasive procedures. At the same time, prospective evidence linking AI classifications to cytology, surgical histopathology, and biopsy-related decision making remains comparatively limited [19,20,21,22,23,24,25,26,27,28,29,30,31,32].

Therefore, the present prospective observational study aimed to compare AI-assisted ACR TI-RADS assessment with routine radiologist-assigned ACR TI-RADS in consecutive thyroid nodules undergoing ultrasound-guided FNA. AI-assisted risk classification was evaluated in relation to Bethesda cytology and, where available, postoperative histopathology. In addition to conventional diagnostic performance, we investigated the degree of discordance between AI and radiologist risk assignments, particularly among cytologically benign nodules, and explored whether AI-assisted assessment might have the potential to refine biopsy selection. Because all nodules included in the study had already undergone FNA, the study was designed to estimate the potential impact on biopsy decision-making rather than to measure an actual reduction in performed biopsies [33,34,35].

## 2. Materials and Methods

This prospective observational study was conducted at the Department of Endocrinology and Metabolism, İnönü University Turgut Özal Medical Center, Malatya, Türkiye. Consecutive adult patients referred for ultrasound-guided fine-needle aspiration (FNA) of thyroid nodules between May 2025 and May 2026 were prospectively screened. The unit of analysis was the thyroid nodule. Each included nodule was derived from a different patient; therefore, the final cohort comprised 1009 nodules from 1009 unique patients, with no patient contributing more than one nodule to the analysis. A total of 1257 nodules were initially assessed. Nodules were excluded if cytological evaluation was nondiagnostic (Bethesda I), ultrasound images were inadequate for AI-assisted assessment, or clinical data required for the planned analyses were incomplete. After these exclusions, 1009 thyroid nodules constituted the final analytic cohort.

Thyroid ultrasound examinations were performed as part of routine clinical care. Target nodules were evaluated according to the ACR TI-RADS framework, including composition, echogenicity, shape, margins, and echogenic foci, together with nodule dimensions. Doppler imaging was also obtained as part of the routine ultrasound examination. The ACR TI-RADS category assigned during routine radiological assessment was recorded independently of the subsequent AI-assisted assessment, and radiologists had no access to the AI-generated results.

AI-assisted assessment was performed using ChatGPT Plus (https://chatgpt.com, accessed on 1 September 2026, OpenAI, San Francisco, CA, USA) through an individual single-user subscription. The AI system was not specifically trained, fine-tuned, or otherwise adapted using images or clinical data from the present study before or during the investigation. During ultrasound-guided FNA, the investigator performing the AI assessment (R.K.) personally submitted representative ultrasound images of the target nodule to ChatGPT Plus. Submitted material included images demonstrating nodule morphology and dimensions as well as Doppler images. Image selection was performed by the investigator at the time of the biopsy procedure rather than automatically by the AI system. The radiologist-assigned TI-RADS category was not disclosed to ChatGPT, and cytological and postoperative histopathological results were unavailable at the time of AI assessment. Thus, the AI assessment was performed independently and without knowledge of either the routine radiologist classification or the reference-standard outcomes.

AI-assisted assessments were performed prospectively between May 2025 and May 2026 and contemporaneously with ultrasound-guided FNA as consecutive nodules were evaluated. For each nodule, a new ChatGPT conversation was initiated to prevent carryover of conversational context from previously assessed nodules. For each nodule, the investigator manually selected the ultrasound image in which the target nodule was most clearly visualized, with dimensional measurements displayed on the image, and uploaded it for AI-assisted assessment. The AI was prompted to predict the ACR TI-RADS category, Bethesda category, and malignancy risk for each nodule. A fixed verbatim prompt template was not prospectively archived; therefore, the exact wording used for each assessment cannot be reproduced retrospectively. However, the standardized task requested in each new conversation was to evaluate the submitted thyroid nodule images and provide an ACR TI-RADS category, a predicted Bethesda category, and an estimated malignancy risk. A corresponding Doppler image of the same nodule was subsequently provided as additional imaging information. Image selection was therefore investigator-dependent rather than automated or based on a predefined image-selection algorithm.

ChatGPT was used as an investigational decision-support tool to reassess the sonographic characteristics of each target nodule, generate a TI-RADS category, and estimate malignancy risk. The exact underlying ChatGPT model version used during the study period was not prospectively recorded. The model’s original training dataset and its provenance were not available to the investigators. However, no patient images, clinical data, or other study-specific material from the present cohort were used by the investigators to train or fine-tune the model. Accordingly, potential overlap between the model’s original pretraining data and the present study cohort could not be independently assessed. ChatGPT Plus was used as an investigational AI-assisted decision-support tool and not as a dedicated CE-marked or FDA-cleared thyroid ultrasound computer-aided diagnostic device.

Both routine radiologist assessment and AI-assisted assessment were performed according to the American College of Radiology Thyroid Imaging Reporting and Data System (ACR TI-RADS). For each AI-assisted evaluation, ChatGPT was specifically instructed to assess the submitted ultrasound images according to the ACR TI-RADS framework. Thus, the AI and radiologist classifications were based on the same risk-stratification system, allowing direct paired comparison. For the prespecified comparative analyses, ACR TI-RADS categories were dichotomized into low-risk (TR2–TR3) and high-risk (TR4–TR5) groups. The comparison between AI and radiologist classifications was intended to quantify discordance in risk assignment rather than to define either assessment as intrinsically correct or incorrect. Because all included nodules had already undergone FNA, differences in high-risk classification were interpreted as estimates of how AI-assisted reassessment might have altered biopsy selection rather than as an observed reduction in biopsies.

Ultrasound-guided FNA was performed according to routine institutional clinical practice. Cytological specimens were interpreted according to the 2023 Bethesda System for Reporting Thyroid Cytopathology and categorized as Bethesda I–VI. Bethesda I nodules were excluded from the final analytic cohort because nondiagnostic cytology could not provide an adequate cytological comparator. Cytology was available for the entire analytic cohort and was used to examine the relationship between ultrasound risk classification and Bethesda category. For patients who subsequently underwent thyroid surgery for clinical indications, definitive postoperative histopathological diagnosis was recorded and served as the reference standard for diagnostic performance analyses. Histopathological verification was available for 73 nodules, including 48 malignant and 25 benign lesions. Because surgery was clinically determined rather than mandated by the study protocol, histopathological verification was not available for all nodules. Diagnostic performance estimates based on histopathology were therefore restricted to the surgically verified subgroup and should not be interpreted as population-level performance estimates for the entire cohort.

The primary clinical analysis compared ACR TI-RADS–based FNA recommendations derived from AI-assisted and radiologist-assigned categories after applying category-specific size thresholds to each nodule. Diagnostic performance against postoperative histopathology was evaluated as a secondary analysis in the surgically verified subgroup, using TR4–TR5 as positive and TR2–TR3 as negative for both approaches. A secondary outcome was discordance between AI-assisted and radiologist-assigned TI-RADS risk categories across the entire cohort and within individual Bethesda categories. Particular attention was given to the number of Bethesda II nodules assigned to TR4–TR5 by each method. To assess whether radiologist-assigned high-risk categories represented guideline-concordant indications for biopsy, ACR TI-RADS categories were evaluated together with the maximum nodule diameter. Guideline-concordant FNA was defined according to the ACR TI-RADS size thresholds: ≥25 mm for TR3, ≥15 mm for TR4, and ≥10 mm for TR5 nodules. For each nodule, these category-specific size thresholds were applied separately to the AI-assisted and radiologist-assigned ACR TI-RADS categories to determine whether FNA would have been recommended by each assessment approach. TR2 nodules were considered not to meet criteria for FNA.

Because all included nodules had undergone FNA, differences between AI and radiologist risk assignments were considered estimates of the potential impact of AI-assisted assessment on biopsy decision-making and were not interpreted as biopsies actually avoided during the study.

Statistical analyses were performed using IBM SPSS Statistics version 29.0 (IBM Corp., Armonk, NY, USA). Continuous variables are presented as mean ± standard deviation and categorical variables as number and percentage. The normality of continuous variables was assessed using the Shapiro–Wilk test. Continuous variables were compared using the independent-samples t test when parametric assumptions were satisfied and the Mann–Whitney U test when these assumptions were not satisfied. Categorical variables were compared using the χ^2^ test or Fisher’s exact test, as appropriate. Because AI-assisted and radiologist assessments represented paired evaluations of the same nodules, differences in both dichotomized low-risk versus high-risk classification and ACR TI-RADS–based FNA recommendations were evaluated using the exact McNemar test. Because each patient contributed only one nodule to the analysis, all observations were independent at the patient level and no adjustment for within-patient clustering was required. Diagnostic performance against postoperative histopathology was evaluated in the surgically verified subgroup. For both AI-assisted and radiologist-assigned ACR TI-RADS classifications, TR4–TR5 was considered a positive test and TR2–TR3 a negative test. Sensitivity, specificity, positive predictive value (PPV), negative predictive value (NPV), and overall accuracy were calculated separately for each assessment approach using postoperative histopathology as the reference standard. For ROC analysis, the original ordinal ACR TI-RADS categories were used as the test variable separately for AI-assisted and radiologist-assigned assessments, with postoperative histopathology (benign versus malignant) as the reference standard, and the area under the curve (AUC) was calculated for each approach. The study was approved by the İnönü University Health Sciences Scientific Research Ethics Committee before initiation of participant enrollment (Decision No. 2025/9444; 10 March 2025; Session No. 5). The study was conducted in accordance with the ethical principles of the Declaration of Helsinki. Written informed consent was obtained from all participants prior to enrollment.

## 3. Results

A total of 1009 consecutive thyroid nodules that underwent ultrasound-guided fine-needle aspiration (FNA) between May 2025 and May 2026 were prospectively included in the study. The mean patient age was 51.5 ± 13.4 years, and 814 patients (80.7%) were female. The mean serum free thyroxine (fT4) level was 0.86 ± 0.22 ng/dL, while the mean thyroid-stimulating hormone (TSH) level was 3.41 ± 2.39 mIU/L (Table 1). The marked female predominance is consistent with the demographic pattern commonly observed in thyroid nodule clinics. The overall cohort also included a broad range of nodule dimensions, with mean width and height of 28.4 ± 8.6 mm and 20.5 ± 7.7 mm, respectively (Table 1).

**Figure 1:**
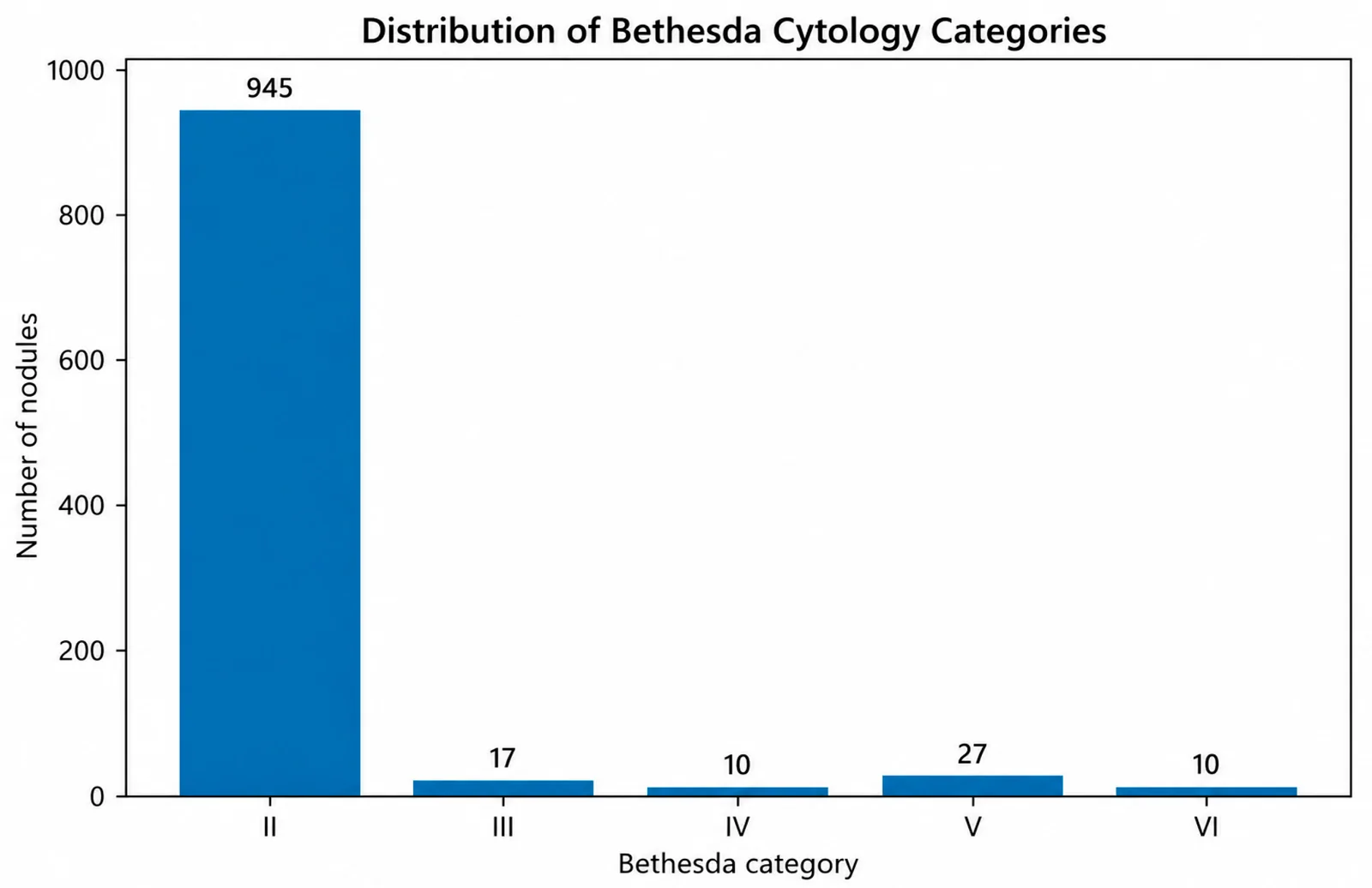
Distribution of thyroid nodules according to the 2023 Bethesda System for Reporting Thyroid Cytopathology.

According to cytological evaluation, Bethesda II was the predominant category, accounting for 945 of 1009 nodules (93.6%), whereas Bethesda III, IV, V, and VI comprised 17 (1.7%), 10 (1.0%), 27 (2.7%), and 10 (1.0%) nodules, respectively (Figure 1).

**Figure 2:**
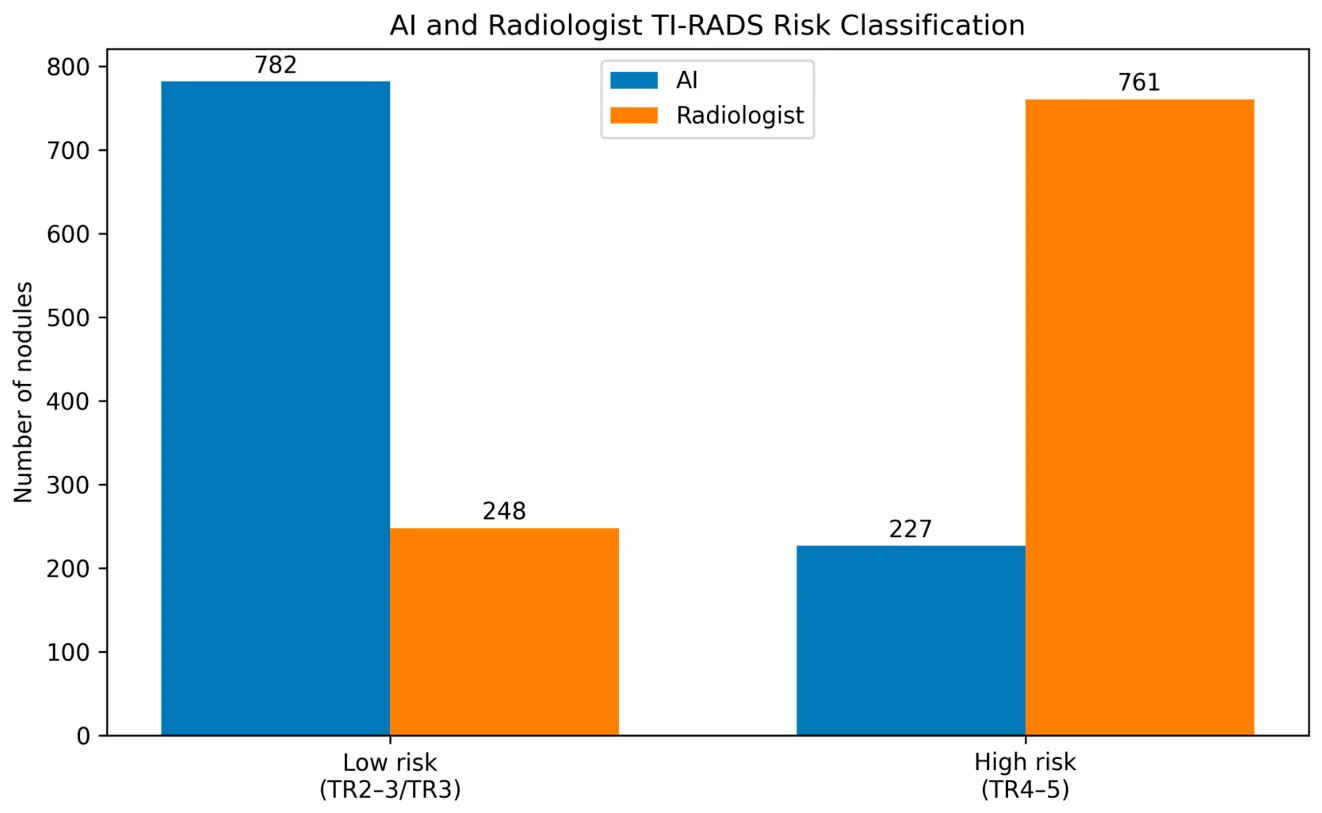
Comparison of low-risk (TR2–TR3) and high-risk (TR4–TR5) classifications assigned by the artificial intelligence-assisted system and radiologists.

The AI-based TI-RADS system classified 782 nodules (77.5%) as low risk (TR2–TR3) and 227 nodules (22.5%) as high risk (TR4–TR5). In contrast, radiologist-assigned TI-RADS categorized only 248 nodules (24.6%) as low risk, whereas 761 nodules (75.5%) were classified as high risk. Overall, AI assigned significantly fewer nodules to the high-risk category than conventional radiologist assessment (exact McNemar test, *p* < 0.001) (Table 2, Figure 2). Thus, the absolute difference in the proportion of nodules labeled high risk by the two approaches was 52.9 percentage points. The direction of this difference was consistent with the study hypothesis that AI-supported classification could reduce escalation to high-risk categories in a cohort already selected for FNA.

To complement the histopathology-based diagnostic performance analysis, a cytology-based subgroup analysis was performed across Bethesda categories in the entire cohort. Among Bethesda II nodules, AI classified 773 of 945 lesions (81.8%) as low risk and only 172 (18.2%) as high risk. In comparison, radiologist TI-RADS classified only 244 nodules (25.8%) as low risk, while 701 nodules (74.2%) were categorized as TR4–TR5 (*p* < 0.001). Similarly, AI assigned fewer Bethesda III nodules to the high-risk category than radiologist assessment (76.5% vs. 88.2%), although this difference was not statistically significant because of the small sample size. All Bethesda V and VI nodules were classified as high risk by the AI system, indicating complete identification of cytologically high-risk lesions (Table 2). The largest divergence between AI-assisted and radiologist-assigned risk categories was observed among Bethesda II nodules. Because the study cohort consisted exclusively of nodules that had already been selected for FNA, this difference should not be interpreted as evidence that the radiologist classifications were incorrect or that the corresponding biopsies were unnecessary; rather, it demonstrates substantial discordance between the two risk-assessment approaches in an FNA-selected population. By contrast, agreement at the high-risk end of the cytological spectrum was complete for Bethesda V and VI.

When ACR TI-RADS category-specific size thresholds were applied to all 1009 nodules, FNA would have been recommended for 469 nodules (46.5%) according to the AI-assisted classification and for 993 nodules (98.4%) according to the radiologist-assigned classification (exact McNemar test, *p* < 0.001). In paired analysis, 524 nodules met FNA criteria according to the radiologist assessment but not according to the AI-assisted assessment, whereas no nodule met FNA criteria according to the AI assessment alone. Thus, AI-assisted classification was associated with 524 fewer modeled FNA recommendations, corresponding to a 52.8% relative reduction compared with radiologist-based recommendations. Because all nodules in the cohort had already undergone FNA, these findings represent a modeled difference in guideline-based biopsy recommendations rather than biopsies actually avoided during the study.

To determine whether the high-risk classifications assigned by radiologists represented guideline-concordant indications for FNA, radiologist-assigned ACR TI-RADS categories were evaluated together with nodule size using the corresponding ACR TI-RADS biopsy thresholds. Among the 761 nodules classified as TR4–TR5 by radiologists, 748 (98.3%) met the size-based ACR TI-RADS criteria for FNA, whereas 13 (1.7%) did not meet the corresponding size threshold. Similarly, among the 701 Bethesda II nodules classified as TR4–TR5 by radiologists, 689 (98.3%) met ACR TI-RADS size criteria for FNA. Thus, the marked difference between radiologist and AI high-risk assignments should not be interpreted as evidence that the biopsies performed following routine radiological assessment were generally inconsistent with established ACR TI-RADS recommendations.

Definitive postoperative histopathological diagnoses were available for 73 nodules, including 48 malignant and 25 benign lesions. Malignant nodules had significantly smaller width (24.46 ± 16.52 mm vs. 36.00 ± 15.96 mm, *p* = 0.002) and height (18.81 ± 13.84 mm vs. 26.88 ± 15.72 mm, *p* = 0.010) than benign nodules. Serum TSH levels were also significantly lower in the malignant group (2.93 ± 2.12 vs. 3.78 ± 2.52 mIU/L, *p* = 0.043), whereas age, sex, and serum free T4 levels were comparable between groups. Ultrasound findings associated with malignancy included predominantly solid composition, hypoechogenicity, microcalcifications, and irregular margins (all *p* < 0.001), while macrocalcification showed no significant association with malignancy (*p* = 0.727) (Table 3). Within this surgically verified subgroup, 65.8% (48/73) of nodules were malignant, demonstrating the expected enrichment of malignancy among patients selected for surgery. This enrichment is important when interpreting PPV, NPV, and overall accuracy. Histopathological verification was therefore available for 73 of 1009 nodules (7.2%). Because surgery was performed according to clinical indications rather than the study protocol, the surgically verified subgroup was enriched for suspicious disease. Accordingly, histopathology-based diagnostic performance estimates were restricted to this subgroup and were not assumed to represent the entire FNA cohort.

Among the 17 Bethesda III nodules, postoperative histopathology was available for 10 lesions, including eight malignant and two benign nodules. All eight malignant Bethesda III nodules were classified as high risk (TR4–TR5) by AI. None of the four Bethesda III nodules classified as AI low risk (TR2–TR3) underwent surgery; therefore, definitive histopathological verification was unavailable for these lesions. Among the 10 Bethesda IV nodules, five were classified as AI low risk, and all five underwent surgery and were histopathologically benign. Of the remaining five Bethesda IV nodules classified as AI high risk, two were malignant and three were benign. Overall, among the 73 surgically verified nodules, 18 were classified as AI low risk and all 18 were histopathologically benign, whereas the AI high-risk group comprised seven benign and 48 malignant nodules. Thus, no histologically malignant nodule in the surgically verified subgroup was classified as AI low risk.

**Figure 3:**
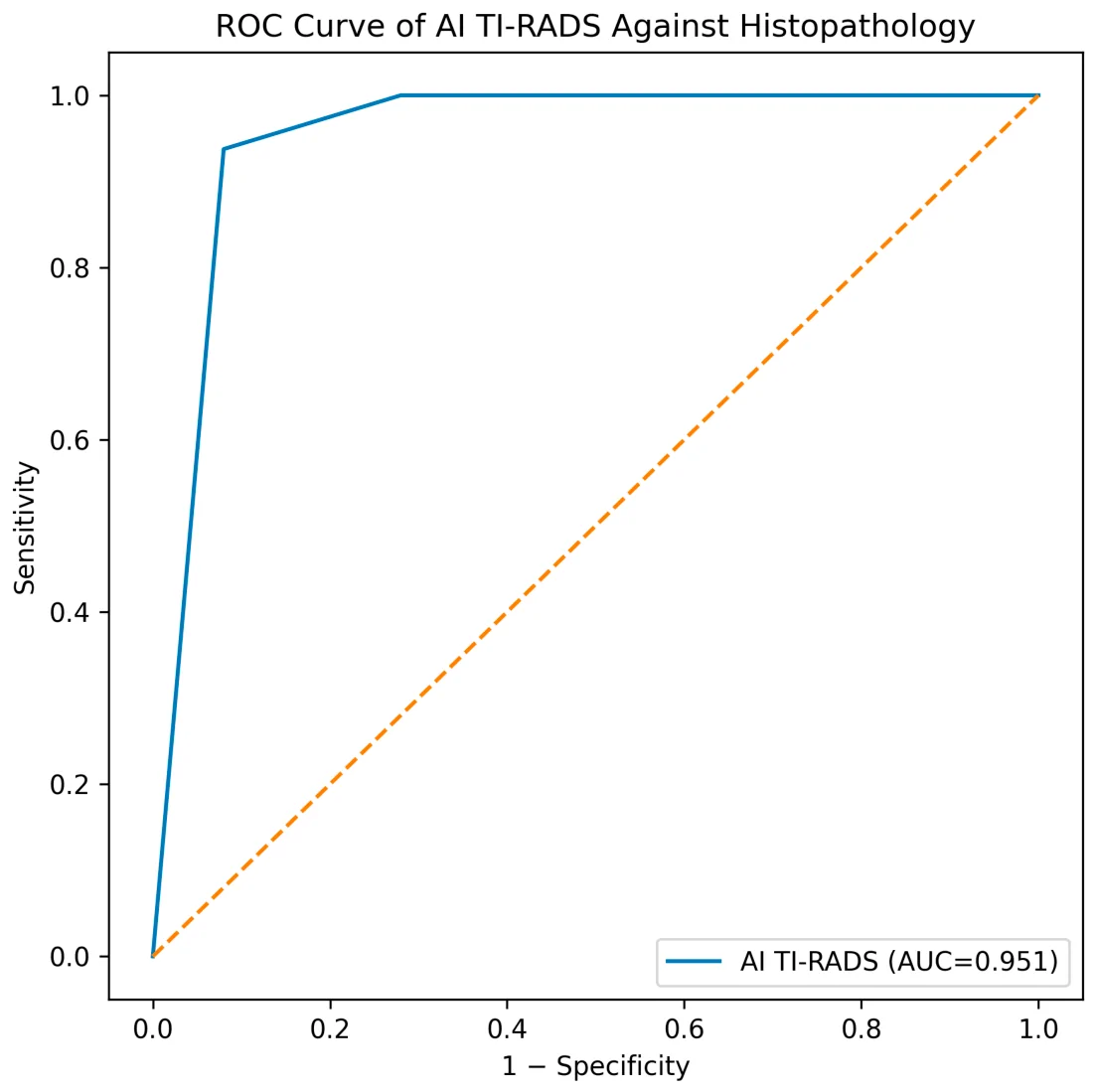
Receiver operating characteristic curve of the ordinal AI-assigned ACR TI-RADS category (TR2–TR5) for discrimination between benign and malignant postoperative histopathology in the surgically verified subgroup (*n* = 73). The area under the curve was 0.951 (95% CI, 0.904–0.998). The orange dashed line represents the reference line (AUC = 0.5). ROC, receiver operating characteristic; CI, confidence interval.

In the surgically verified subgroup, diagnostic performance was evaluated using the same ACR TI-RADS–based definition for both AI-assisted and radiologist-assigned classifications, with TR4–TR5 considered positive and TR2–TR3 negative. Both approaches correctly classified all 48 histopathologically malignant nodules as high risk, corresponding to a sensitivity of 100.0% and an NPV of 100.0% for both methods. AI-assisted TI-RADS demonstrated a specificity of 72.0%, PPV of 87.3%, and overall accuracy of 90.4%, compared with 60.0%, 82.8%, and 86.3%, respectively, for radiologist-assigned TI-RADS. ROC analysis using the original ordinal TI-RADS categories yielded an AUC of 0.951 for AI-assisted assessment and 0.932 for radiologist assessment (Table 4, Figure 3).

**Figure 4:**
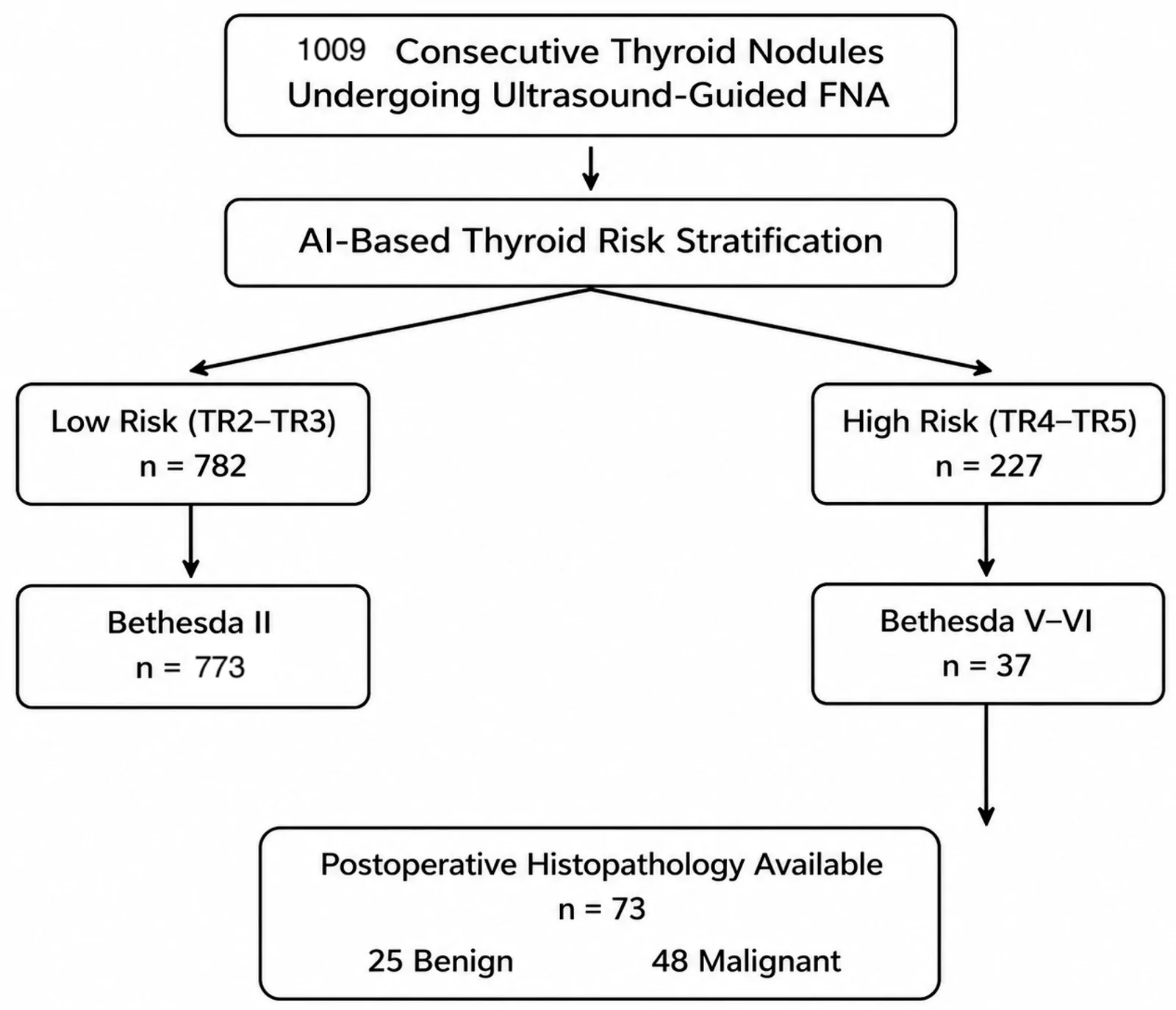
Study flow illustrating AI-based thyroid nodule risk stratification, distribution of low- and high-risk nodules, and availability of postoperative histopathological evaluation.

Finally, the potential clinical impact of AI-based risk stratification was evaluated by comparing biopsy-related high-risk classification with conventional radiologist assessment. AI classified only 227 nodules (22.5%) as high risk, compared with 761 nodules (75.5%) by radiologist TI-RADS (*p* < 0.001). Among cytologically benign (Bethesda II) nodules, AI classified 172 lesions (18.2%) as high risk, whereas radiologist assessment classified 701 lesions (74.2%) as high risk, corresponding to 529 fewer high-risk classifications, an absolute reduction of 56 percentage points, and a relative reduction of 75.4%. All 37 Bethesda V–VI nodules were classified as high risk by the AI-assisted assessment (Figure 4, Table 5). However, this finding should not be interpreted as evidence that AI-assisted biopsy de-escalation would preserve malignancy detection across the entire cohort, because histopathological verification was limited to the surgically treated subgroup.

## 4. Discussion

In this prospective study of 1009 consecutive thyroid nodules undergoing FNA, the AI-assisted ultrasound system demonstrated two findings with direct clinical relevance. First, in the surgically verified subgroup, both AI-assisted and radiologist-assigned ACR TI-RADS achieved 100% sensitivity, while AI-assisted assessment showed higher specificity (72.0% vs. 60.0%) and accuracy (90.4% vs. 86.3%). The corresponding AUCs were 0.951 for AI-assisted assessment and 0.932 for radiologist assessment. The difference was most pronounced among Bethesda II nodules, in which the number of high-risk classifications fell from 701 to 172. At the same time, all Bethesda V–VI nodules remained in the AI high-risk group. These findings suggest that the potential value of AI lies not only in identifying malignancy but also in improving the balance between sensitivity and specificity at the point where ultrasound risk stratification influences invasive investigation [21,22,23,24,25,26].

Ultrasound remains the cornerstone of thyroid nodule evaluation, yet it is inherently operator dependent. Standardized systems such as ACR TI-RADS, EU-TIRADS, and K-TIRADS were developed in part to reduce this variability, but application of their component features still requires subjective judgments [5,6,7,8,9,10,11,12,13]. A mildly hypoechoic nodule with an equivocal margin or punctate echogenic foci, for example, may be scored differently by readers with different levels of experience. Such differences become clinically important when a small change in score moves a nodule across a category or size-specific biopsy threshold. In our cohort, radiologists classified 75.5% of nodules as TR4–TR5, compared with 22.5% by AI. Because the cohort consisted entirely of nodules that underwent FNA, this finding does not prove that three quarters of biopsies were unnecessary; rather, it demonstrates substantial divergence in risk assignment and raises the possibility that AI could serve as a second reader when conventional assessment appears disproportionately suspicious.

Recent advances in deep learning have led to rapid development of AI algorithms for thyroid ultrasound interpretation. Multicenter diagnostic studies, computer-aided systems, and systematic reviews have generally reported encouraging sensitivity and specificity, although performance varies according to dataset composition, reference standard, image acquisition, and model architecture [6,7,8,9,10,11,19,20,21,22,23,24]. Recent prospective studies have further supported the potential clinical utility of AI-assisted thyroid ultrasound assessment. In a prospective study of solid thyroid nodules, AI-assisted ultrasound was evaluated for diagnostic performance and its potential to reduce unnecessary biopsy, while a large multicenter prospective real-world study demonstrated the feasibility of AI assistance across radiologists with different levels of experience [36,37]. A recent review of AI applications in thyroid ultrasound similarly emphasized that limited generalizability, heterogeneous imaging conditions, and the lack of large-scale real-world validation remain major barriers to clinical translation, highlighting the need for rigorous multicenter external validation [38]. The literature has also shifted from proof-of-concept classification toward questions of clinical implementation. This is an important transition: a system may achieve high diagnostic accuracy in a curated image set but still fail to improve decisions in real-world practice. Our study contributes to this implementation-focused evidence by evaluating consecutive nodules prospectively and by examining the relationship between AI risk assignment, routine radiologist classification, Bethesda cytology, and surgical histopathology within the same clinical pathway.

The most pronounced discordance was observed among Bethesda II nodules. Of 945 cytologically benign nodules, 701 (74.2%) were categorized as TR4–TR5 by routine radiologist assessment compared with 172 (18.2%) by AI, corresponding to 529 fewer high-risk assignments. Importantly, this difference should not be interpreted as evidence of inappropriate radiologist-guided biopsy. When radiologist-assigned ACR TI-RADS categories were evaluated together with the corresponding size thresholds for FNA, 748 of 761 (98.3%) radiologist-classified TR4–TR5 nodules met guideline-based size criteria for biopsy. Among Bethesda II nodules classified as TR4–TR5 by radiologists, 689 of 701 (98.3%) likewise met the ACR TI-RADS size criteria for FNA. Furthermore, a Bethesda II result after FNA does not retrospectively establish that the preceding biopsy was unnecessary, because ACR TI-RADS is a pre-test risk-stratification system and benign cytology is an expected outcome of many guideline-concordant biopsies. The observed difference should therefore be interpreted as discordance between two risk-assessment approaches rather than radiologist error. Nevertheless, the substantially lower frequency of high-risk assignments by AI raises the hypothesis that AI-assisted reassessment could help refine future biopsy-selection strategies, a possibility that requires prospective testing before clinical implementation.

Preservation of sensitivity is essential before any AI-assisted strategy for biopsy de-escalation can be considered clinically safe. In the present study, all 37 Bethesda V–VI nodules were assigned to the high-risk group by AI, and all 48 histologically malignant nodules within the surgically verified subgroup were identified by the binary AI prediction. However, these findings should not be interpreted as demonstrating absence of missed malignancy across the entire cohort. Histopathological verification was available for only 73 of 1009 nodules (7.2%), and the decision to proceed to surgery was clinically driven. This creates partial verification bias because nodules with suspicious cytology, imaging findings, symptoms, or other clinical indications were more likely to undergo definitive histopathological assessment. Conversely, nonoperated nodules, including those assigned to the AI low-risk group, were predominantly verified by cytology rather than surgical histopathology. Consequently, a malignancy occurring in an AI-low-risk nodule that did not undergo surgery could not be definitively excluded by the present study design. The observed 100% sensitivity should therefore be interpreted strictly as performance within the surgically verified subgroup rather than evidence that AI-guided biopsy de-escalation is oncologically safe.

Our histopathology-based AUC of 0.951 is also compatible with the upper range of performance reported in contemporary AI literature. A 2025 systematic review and meta-analysis of ultrasound-feature-based AI models reported pooled sensitivity of 0.89, specificity of 0.84, and a summary AUC of 0.93 across 28 studies, while emphasizing substantial heterogeneity between models and study designs. Recent studies specifically addressing biopsy decisions have likewise suggested that combining AI with conventional TI-RADS can reduce unnecessary FNA, although the magnitude of benefit and the risk of missed malignancy differ between systems and populations. These comparisons reinforce the need to evaluate each AI platform in its intended clinical setting rather than assuming interchangeability among algorithms [39,40].

An additional point concerns the interaction between AI and the human operator. AI output is not independent of image acquisition: probe position, image plane, nodule segmentation, and selection of representative images can influence the features available to an algorithm. A recent clinical trial of human–AI collaboration showed substantial variation in AI diagnostic accuracy despite use of the same ultrasound systems, illustrating that operator effects can persist even after an AI tool is introduced. For this reason, AI should be viewed as part of a human–machine workflow. Standardized image acquisition, appropriate training, and clinician review of discordant cases are likely to remain essential even when automated risk estimates are available.

The discordance observed in our cohort may therefore be clinically useful rather than simply interpreted as competition between AI and radiologists. A practical implementation model could use AI as an independent second reader: concordant high-risk assessments could reinforce the indication for biopsy, concordant low-risk assessments could support surveillance when size criteria allow, and discordant assessments could trigger targeted review of the sonographic features responsible for the discrepancy. Such a workflow preserves physician responsibility while using AI to identify cases in which subjective interpretation may have inflated or underestimated risk. Importantly, our data do not establish a new biopsy threshold and should not be used to override established guideline criteria without prospective validation.

The distribution of the study population also deserves consideration. Bethesda II accounted for 93.6% of the cohort, reflecting the predominance of benign cytology among nodules selected for FNA. This distribution provides a large sample for studying overclassification in benign nodules, but it also means that the numbers in Bethesda III and IV were small. The observed 8 malignant tumors among the 10 surgically treated Bethesda III nodules should not be interpreted as an 80% malignancy rate for all Bethesda III nodules, because surgery was performed in a selected subgroup with additional clinical indications. The same verification process explains the high malignancy prevalence of 65.8% in the overall surgical subgroup and limits direct generalization of PPV and NPV to an unselected thyroid nodule population.

The study has several strengths. The prospective design and consecutive inclusion reduce some of the selection and information biases associated with retrospective image-bank studies. The cohort of 1009 nodules is sufficiently large to characterize differences in risk assignment across the common Bethesda categories, particularly Bethesda II. Cytology was standardized using the 2023 Bethesda System, radiologists were blinded to AI output, and postoperative histopathology was available as a definitive reference in surgically treated nodules. In addition, the study examined a clinically meaningful endpoint—potential reduction in high-risk classification—rather than limiting evaluation to AUC or accuracy. The use of paired AI and radiologist assessments on the same nodules also allows the observed difference in risk categorization to be interpreted without between-cohort confounding.

Several limitations should be acknowledged. First, this was a single-center study performed in a tertiary referral setting, and the patient spectrum, referral patterns, radiologist experience, and prevalence of suspicious nodules may differ from those in community practice or screening populations. Second, histopathological confirmation was available only for surgically treated nodules. This partial verification introduces selection bias because surgery is more likely in patients with suspicious cytology, ultrasound findings, symptoms, or other clinical indications; consequently, the 65.8% malignancy prevalence in the surgical subgroup is substantially enriched compared with the overall cohort. Diagnostic predictive values from this subgroup should therefore not be interpreted as population prevalence estimates. Third, Bethesda II cytology was used as the principal marker of benignity in the analysis of potentially unnecessary high-risk classification. Although Bethesda II carries a low expected risk of malignancy, cytology is not equivalent to surgical histology, and long-term follow-up would strengthen confirmation of benign outcomes. In addition, systematic longitudinal ultrasound or histopathological follow-up was not available for all nonoperated AI-low-risk nodules. Therefore, although Bethesda II cytology provides strong evidence of benignity, the possibility of cytological false-negative disease in this group cannot be completely excluded. This limitation is particularly important when considering the potential use of AI to de-escalate biopsy recommendations and prevents the present study from establishing the safety of such a management strategy. Fourth, all nodules included in this study had already been selected for FNA in routine clinical practice, resulting in an FNA-enriched cohort rather than an unselected thyroid nodule population. This selection may have altered the distribution of TI-RADS categories and malignancy risk and limits the generalizability of our findings to unselected screening populations or nodules that would not ordinarily undergo biopsy. Accordingly, the observed differences in risk classification and modeled FNA recommendations should not be interpreted as a directly observed reduction in biopsy procedures. These findings should be validated prospectively in broader, unselected populations before AI-assisted assessment can be considered for biopsy-de-escalation strategies. The cohort was also predominantly female, reflecting a sex-imbalanced study population. Although ACR TI-RADS classification is based primarily on sonographic nodule characteristics rather than patient sex, the present study was not designed or powered to determine whether AI-assisted classification performance differs by sex. Therefore, the generalizability of the findings to more sex-balanced populations should be confirmed in future studies. Fifth, only one AI platform was evaluated, and algorithm performance may vary according to software version, training data, ultrasound equipment, image acquisition, and operator interaction. In addition, the ultrasound images submitted for AI-assisted assessment were manually selected by a single investigator based on the clearest visualization of the target nodule. This investigator-dependent image-selection process represents a potential source of operator-related variability. Because independent repeat image selection was not performed, the reproducibility of AI classifications across different image selections or operators could not be assessed. Future studies should incorporate standardized or automated image-selection procedures and evaluate inter-operator reproducibility. Finally, Interobserver variability was not directly assessed in the present study. Each nodule was evaluated within the routine clinical workflow without an independent second radiologist reading, and repeated AI assessments were not systematically performed. Therefore, although interobserver variability in ultrasound-based TI-RADS classification and the potential reproducibility of AI-assisted assessment are discussed in the context of previous literature, the present study does not provide direct comparative data on human interobserver agreement or AI test–retest reproducibility. Future studies incorporating multiple independent radiologist readers and repeated AI assessments are needed to determine whether AI-assisted evaluation improves reproducibility relative to conventional ultrasound interpretation.

## 5. Conclusions

In conclusion, AI-assisted ACR TI-RADS assessment produced substantially fewer high-risk assignments than routine radiologist assessment in this prospective single-center cohort of thyroid nodules undergoing FNA, with the greatest discordance observed among Bethesda II nodules. These findings support further investigation of AI as an independent second-reader decision-support tool that may help refine thyroid nodule risk stratification and biopsy selection. However, because the cohort consisted exclusively of nodules that had already undergone FNA and histopathological verification was limited to 73 surgically treated nodules, the present study cannot establish that AI-guided biopsy de-escalation is safe or that malignancy would not be missed among nonoperated AI-low-risk nodules. Prospective management studies incorporating AI before the decision to perform FNA, together with systematic longitudinal or histopathological verification, are required to determine whether this approach can safely reduce unnecessary biopsies while preserving cancer detection.

## Figures and Tables

**Table 1:** Baseline Demographic and Clinical Characteristics.

Variable	Overall Cohort (*N* = 1009)
Age, years	51.5 ± 13.4
Female sex, *n* (%)	814 (80.7)
Male sex, *n* (%)	195 (19.3)
Nodule width, mm	28.4 ± 8.6
Nodule height, mm	20.5 ± 7.7
Free T4, ng/dL	0.86 ± 0.22
TSH, mIU/L	3.41 ± 2.39

**Table 2:** Comparison of AI-Based and Radiologist TI-RADS Across Bethesda Categories.

Bethesda	*n*	AI TR2–3	AI TR4–5	Radiologist TR2–3	Radiologist TR4–5	*p*
II	945	773 (81.8)	172 (18.2)	244 (25.8)	701 (74.2)	<0.001
III	17	4 (23.5)	13 (76.5)	2 (11.8)	15 (88.2)	0.625
IV	10	5 (50.0)	5 (50.0)	2 (20.0)	8 (80.0)	0.375
V	27	0	27 (100.0)	0	27 (100.0)	1.000
VI	10	0	10 (100.0)	0	10 (100.0)	1.000
Total	1009	782 (77.5)	227 (22.5)	248 (24.6)	761 (75.5)	<0.001

**Table 3:** Characteristics of Surgically Treated Nodules According to Histopathology.

Variable	Benign (*n* = 25)	Malignant (*n* = 48)	*p*
Age (years)	51.60 ± 15.17	51.42 ± 13.54	0.926
Female sex	20 (80.0)	34 (70.8)	0.575
Nodule width (mm)	36.00 ± 15.96	24.46 ± 16.52	0.002
Nodule height (mm)	26.88 ± 15.72	18.81 ± 13.84	0.010
Free T4	0.82 ± 0.22	0.85 ± 0.20	0.600
TSH	3.78 ± 2.52	2.93 ± 2.12	0.043
Solid composition	7 (28.0)	45 (93.8)	<0.001
Hypoechogenicity	9 (36.0)	47 (97.9)	<0.001
Microcalcification	9 (36.0)	41 (85.4)	<0.001
Irregular margin	1 (4.0)	33 (68.8)	<0.001

**Table 4:** Diagnostic Performance of AI-Assisted and Radiologist-Assigned ACR TI-RADS in the Surgically Verified Subgroup.

Measure	AI-Assisted TI-RADS	Radiologist TI-RADS
Surgically verified nodules, *n*	73	73
Low-risk (TR2–3): benign/malignant	18/0	15/0
High-risk (TR4–5): benign/malignant	7/48	10/48
Malignant nodules classified low-risk	0 (0%)	0 (0%)
Sensitivity	100.0%	100.0%
Specificity	72.0%	60.0%
PPV	87.3%	82.8%
NPV	100.0%	100.0%
Accuracy	90.4%	86.3%
AUC	0.951	0.932

TR4–TR5 was considered a positive test and TR2–TR3 a negative test for both AI-assisted and radiologist-assigned ACR TI-RADS classifications, with postoperative histopathology as the reference standard. Sensitivity, specificity, positive predictive value (PPV), negative predictive value (NPV), and accuracy were calculated using this dichotomization. AUC was calculated using the original ordinal ACR TI-RADS categories. PPV, positive predictive value; NPV, negative predictive value; AUC, area under the receiver operating characteristic curve.

**Table 5:** Clinical Impact of AI on Biopsy Selection.

Outcome	AI	Radiologist	*p*
High-risk classification	227 (22.5%)	761 (75.5%)	<0.001
Low-risk classification	782 (77.5%)	248 (24.6%)	<0.001
Bethesda II classified high risk	172 (18.2%)	701 (74.2%)	<0.001
Bethesda V–VI classified as high risk	37/37 (100%)	37/37 (100%)	1.000
Absolute difference in high-risk classifications, overall cohort	534 fewer	Reference	—
Relative reduction in high-risk classifications, overall cohort	70.2%	Reference	—

## Data Availability

The data supporting the findings of this study are available from the corresponding author upon reasonable request. The data are not publicly available because they contain potentially identifiable patient-level clinical information and are subject to privacy and ethical restrictions related to participant confidentiality.
